# Personal statement versus psychological test as admission to the nursing degree: an evaluation

**DOI:** 10.1186/s12912-022-00919-w

**Published:** 2022-06-17

**Authors:** Marian Traynor, Colin Mc Neill, Audrey Roulston

**Affiliations:** 1 School of Nursing & Midwifery, Queen’s University Belfast, 97 Lisburn Road, Belfast, BT9 7BL N. Ireland; 2 Identity Exploration Ltd, 50 Stranmillis Embankment, Belfast, Co Antrim N. Ireland; 3 School of Social Sciences, Education and Social Work, Queen’s University Belfast, 6 College Park, Belfast, BT7 1NN N. Ireland

**Keywords:** Personal statement, Psychometric test, Psychological test, Pre-registration nursing, Admissions, Nursing student, Values-based recruitment

## Abstract

**Background:**

A review of admissions to nursing in Northern Ireland was prompted by the growing number of applications and a desire to ensure that the applicants had the right values for a career in nursing. Concerns regarding authorship, plagiarism and reliability of personal statements used to select applicants to interview was the focus of this research. This study evaluates the psychometric properties of a Personal Statement (PS) as a method for admission to a nursing programme and a values-based psychological screening tool, Nurse Match (NM).

**Methods:**

A self-selecting, purposive sample (*n* = 228; 9.7%) was drawn from applicants to Schools of Nursing in the United Kingdom (*n* = 2350). Participants all of whom had completed a Personal Statement were asked to complete a psychological tool and the scoring outcomes and psychometric properties of both tests were investigated. Statistical analysis was conducted using Minitab 17.

**Results:**

Applicants from 18 schools and five colleges responded. The majority (72.4%) were aged 18–19. Findings provide practical, theoretical, statistical, and qualitative reasons for concluding that the Personal Statement has substantial limitations as a measure of suitability. It does not compare well with international test standards for psychometric tests. In contrast, NM is a valid and reliable measure with good discriminatory power, standardised administration and consistent marking.

**Conclusion:**

NM is a viable alternative to the PS for shortlisting applicants for nursing interviews.

## Introduction

The application of robust quality assurance procedures to the selection methods used for entry to university is good practice and there is always the need to forward plan and develop new approaches to selection in response to national and regional requirements and evidence-based practice [4, 16, 17, 20]. A further consideration is that within professional courses such as nursing there is a requirement to select individuals that will ultimately become competent and caring practitioners capable of delivering safe and effective patient care. Selection methodologies therefore need to be consistent with this desired outcome.

The recently published Universities and College Admissions Service (UCAS) & Health Education England (HEE) report into nurses of the future, [23] highlights that the nursing profession is becoming a more desirable career pathway for many and consequently application to study nursing is increasing. This is not only within the United Kingdom as internationally, Schools of Nursing have also seen a surge in applications [1]. With this increased interest in nursing as a career choice, comes a parallel increase in responsibility for HEIs to ensure that their selection methodologies are robust. It is important therefore that methods used to select into nursing will withstand internal and external scrutiny and furthermore be able to deal with the challenges from applicants who fail to gain a place. The tests are generally a gate keeping mechanism used to decide who progresses through to interview and the Personal Statement is one test generally used within the UK and more widely [5, 19, 26].

The personal statement is a test or an assessment used for making judgements and therefore there is a need to ensure that the assessment is fair, transparent and as objective as possible. Validity and reliability are therefore fundamental to the rigour of the personal statement as a selection tool especially when decisions based on them, similar to any assessment, are final and have lasting consequences [7, 10–12].

Like all forms of assessment, the personal statement can be problematic, prone to subjectivity by the assessor and open to claims of plagiarism [7, 19]. Despite this it is widely used by many nursing schools, and crucially it is a high stakes assessment which the candidate must pass in order to progress to interview. It is concerning therefore that there appears to have been very little research to address the quality assurance issues so adequately identified by Patterson et al. in the 2018 Ottawa consensus statement [16].

Research nationally and internationally has explored whether personal statements are fit for purpose and the general consensus is that they are not. However, personal statements continue to be used. The main aim of this study was to evaluate the personal statement as a tool for shortlisting applicants to nursing in conjunction with a psychological test as a potential alternative. This quantitative research study was funded by the Burdett Trust for nursing and the development of the psychological test instrument used in the study was previously reported by McNeill et al. [9].

## Background

The health service in the UK is facing many challenges, including deficits that are damaging its reputation for providing professional care and treatment of patients, as well as workforce planning. However, applications to nursing for 2021 saw a 32% increase on 2020 figures. Nursing leaders have attributed the ‘extraordinary leap’ to be partly attributed to inspirational nurses during the Coronavirus pandemic [13]. This increase offers an opportunity to review the assessment of personal values and natural attributes underpinning professional practice, identity, leadership, and teamwork. Selecting applicants, most likely to complete nursing programmes, enhances the quality of patient care, and produces graduates to fill vacancies in the workforce [18]. Selection methods should reliably identify whether candidates are likely to be successful in their training and ultimately become competent clinicians. Evidence shows clearly that academic records are more effective selection methods than traditional interviews, references, and personal statements [14, 15]. Conclusions from a systematic review of nursing programmes [5] support this view and comment that there is insufficient evidence regarding interviews and personal statements. Furthermore, evidence suggests that selecting individuals with personal values mirroring those of the organisation in which they will work, enhances organisational effectiveness [8].

### Personal statements

Currently the Universities and College Admissions Service (UCAS) Personal Statement (PS) submitted by applicants to outline their motivation and commitment to nursing or midwifery, is marked and used by universities to screen and short list for suitability for interview. The information on the UCAS portal [22] describes the PS as open and unstructured and as a line of text limited to 47 lines. According to the UCAS site it is designed to give candidates an opportunity to write about their achievements and their interest in the subject they are applying for, as well as their suitability for, interest in, and commitment to higher education. It is not therefore presented as a test or questionnaire but simply as an opportunity to promote self as an applicant in competition with others.

The UCAS request for a personal statement to accompany an application to an HEI can be regarded as a psychometric personality “test”. It is customary to speak of psychological measurement as a test when it is used primarily to assess some characteristics of an individual. It should therefore conform to the American Psychological Association (APA) test standards. The *standards* cover essential elements in testing including validity, reliability, errors of measurements, and fairness in testing. They also establish standards in relation to testing operations including test design and development, scales and norms, test administration and documentation including score interpretation [2].

However, the derivation of the PS concept and screening criteria being assessed is not well published [3]. The reliability of the PS for shortlisting has been debated nationally and internationally, with consensus that it is not fit for purpose. The 2018 Ottawa consensus statement ‘Selection and recruitment to the healthcare professions’ [16] recommended more evidence-based approaches to selection. Although the PS has high candidate acceptability, it is highly susceptible to plagiarism, coaching or bias, is unreliable and squanders resources [4, 16]. Furthermore, there is a need to establish best practice regarding admission criteria [25].

This study was part of a larger Burdett funded research study that sought to identify applicants to nursing who have the personal values required to build a skilled and competent workforce [21]. This paper reports on the collection of empirical evidence about the psychological characteristics and general effectiveness of the PS to better understand limitations and suggest a viable alternative.

## The study

### Aim

The aim of this study was to collect and evaluate validity evidence for a psychological test (Nurse Match) as a potential selection method and as an alternative to the Personal Statement (PS). This paper will focus on the evaluation of the Personal Statement, validity and reliability and how the scores for the PS compared with the scores from the psychological test.

### Design

The research design was a self-selecting, purposive sample of regional applicants to nursing. A within-subjects repeated measures design was used the dependent variable being suitability for nursing. The volunteers had completed a UCAS PS during their HEI applications. The volunteers were provided with background information to the study and were required to offer their informed consent. The main research activity required them to complete the Nurse Match (NM) instrument online, under formal supervisory conditions. The research used quantitative measurements i.e. two measures of the dependent variable were used, score on the PS and score on NM. A self-selecting sub-sample of personal statements (*n* = 132) were ‘blind’ marked at two regional HEIs.

### Participants

A convenience sample was used by inviting all regional school and colleges, who historically provided applicants to nursing degree programmes (via the UK-wide UCAS system), to participate in the research study. Career’s teachers who responded were briefed on the study. The career teachers then advertised the study to their students who then volunteered to take part and arrangements were made for supervised data collection at the school or college. This resulted in a self-selecting, purposive sample of regional applicants.

Recruitment took place over a 3-month period. The volunteers sat the NM test via an online link in groups supervised by staff in a classroom or computer laboratory. They received a scripted briefing from the supervisor about the process, logging on, use of the software and completion of feedback. The process included reading a Participant Information Sheet (PIS), completion of a Consent Form and reading Instructions for Completing the Test. On completion the test scores, were calculated and stored securely.

Two hundred and twenty-eight (*n* = 228) regional applicants volunteered (*N* = 2350; 9.7%) to take part in the study.

### Data collection

The following sources were used to evaluate the PS and the NM psychological test:

- Participants scores from the PS
- Participants scores from the online NM

The validity, reliability, fairness, and consistency of the tests were assessed together with psychometric probity for compliance with international test standards [2].

### The personal statement

The PS is an integral part of an application for entry to UK Universities. The content of the composition is entirely up to the applicant but is limited to 47 lines of text [22].

The PS is scored by course tutors at the UCAS participant university to which an application is made [22]. The PS Scoring Form used by both HEIs in this study cites four criteria: Personal desire for a career in nursing, Motivation for nursing, Expectations of the course and nursing as a profession, Decision making affecting self and others. The PS is marked out of four in respect to each criterion: maximum score 16. Applicant UCAS ID numbers were used to locate the applicant’s PS scores at regional HEIs.

Participants (*n* = 228) had completed the PS in their own time and were free if they wished to obtain advice and assistance. Academic staff marked the PS (HEI 1 *n* = 196: HEI 2 *n* = 137). A sub-group who had applied to both HEIs (*n* = 132) were ‘double marked’.

### The NM psychological test

NM is custom built from ethnographic nursing data using Ipseus software for test construction and scoring that operationalises well-established identity theory [6, 24]. It measures the extent to which personal values match professional values. Applicants appraise 13 entities from personal, home, and work domains (Table 1) on 20 bi-polar constructs.

**Table 1:** Entities used in the replication study

	Entities ***(incl. aspects of self)***
**01**	**Ideal self**
**02**	**Self at school or work**
**03**	**Self at home**
**04**	**Self under pressure**
**05**	**Me 2 years ago**
**06**	**Me in 5 years’ time**
**07**	**A disliked person**
**08**	**A model nurse**
**09**	**A ward sister**
**10**	**Patients**
**11**	**A bad nurse**
**12**	**My best friend**
**13**	**My parents**

**Fig. 1:**
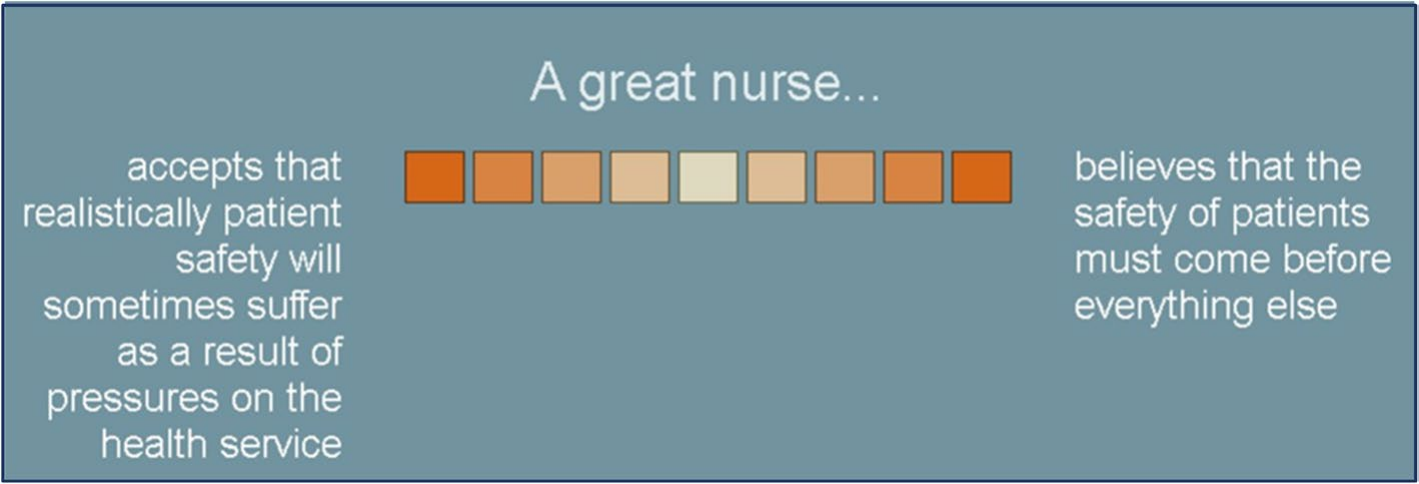
An example of a value construct as presented to applicants

Each bi-polar construct is a value dimension presented on a nine-point semantic differential scale connecting two contrasting perspectives one of which is a preferred professional attitude (Fig. 1). A response scored from 1 to 4 on the point of view it represented. Respondents indicated their preferred (personal) perspective when appraising ‘aspirational self’. The centre zero was used by the respondent if they could not decide between polar values.

Responses are used to calculate a score on each value dimension, each value theme, and an overall score. A quality check is performed on test use.

### Ethical considerations

A research ethics application was submitted to the School of Nursing and Midwifery Research Ethics Committee in December 2017. All ethical issues were properly addressed including information statements and informed consent. Considering the proposed data analysis, participants were asked permission to share their data between the university and the collaborating external company responsible for the NM tool. The anonymisation of the data, the data processing agreement and the confidentiality agreement were also addressed.

Although there was no anticipated risk to the participants they could be perceived as vulnerable as they were all applying for a nursing place and that refusal to participate might impact negatively on their application. Therefore, all communications with potential participants was made via the career teachers and it was made clear that students were under no obligation to volunteer and that involvement in the research would have no effect on their application to nursing. Furthermore, it was made clear that the focus of the study was the evaluation of the NM tool and not individual participants’ performance.

### Data analysis

Descriptive statistics were used to establish and compare group norms for scores. Correlation coefficient *r* and Cronbach’s alpha coefficient were used to investigate the relationship between the scores (Minitab 17).

## Results

Two hundred and twenty-eight (*n* = 228) regional applicants volunteered (*N* = 2350; 9.7%). Eleven of the sample did not provide age data. The 217 who did were aged 16–17 (11); 18–19 (157); 20–24 (26); 25–30 (8); 30+ (15). The majority were female (95.6%: *n* = 218) and aged 18–19 (~ 72.4%).

One early finding was that marking the PS appears to assume that the information provided is valid and reliable whereas the extent of an applicant’s involvement in the composition is uncertain and markers are aware of this. UCAS offers guidance on how to write a personal statement. Applicants are reminded “You’re telling admissions staff why you are suitable to study at their university or college” [22]. During the research, stakeholders shared, uninvited, anecdotal evidence about parents and schools searching for further guidance, the quality and extent of which was wealth dependent. If true, and there was no evidence to suggest otherwise, it is difficult to be sure how much reliable knowledge about an applicant’s potential is being gleaned from their PS. Furthermore, it was unclear which overall concept was being addressed. Nowhere is this stated clearly so we assumed it to be ‘Suitability for Nursing’.

Results from this study suggest that the four criteria used to mark the PS (Desire, Motivation, Expectations, Decision Making) are appropriate and there is statistical evidence to suggest, via correlation and factor analysis, that a quite coherent (unidimensional) overall concept exists (Table 8 below for details). However, while these criteria are described they are not clearly defined, to the detriment of the marking task.

Does the PS test measure what it purports to measure? The results indicate that there are many questions to answer about the use of the PS in screening for interview or selection of candidates: that is regarding content and construct validity, credibility, fitness, robustness, reliability, integrity, representativeness, coherence, and transparency. These concerns are expressed in greater detail in Table 2.

**Table 2:** Summary of evidence about validity of the Personal Statement as a test

Validity	What is the evidence about validity of the Personal Statement (PS)?
***Classic Concepts***	
**Face validity**	**A close examination of the test criteria reveals some concern about evidence for validity in terms of the three classic concepts of validity. On content validity there is no evidence of judgement by ‘experts’ on the content or relevance of the criteria used. No case is made for their use as against other important criteria. The criteria used appear appropriate on their face but are not well defined.** **On construct validity, the four criteria do seem necessary, appropriate, but no case has been made that they are sufficient, that taken together, they capture the (unspecified) quality of ‘suitability for a nursing career’? They are also broadly conceived and poorly defined making consistent data analysis (marking the criteria) difficult. On the face of it the criteria have been useful. They address some important attributes and there is statistical evidence to suggest, via correlation and factor analysis, that the overall concept assessed is coherent (unidimensional). However, the validity of the concept of using an essay about self in ‘assessment at a distance’ is open to questions about authorship, collaboration, integrity, support, powers of expression in writing etcetera and of subjectivity and unfairness in marking it making it unsatisfactory. The evidence is that the PS can be an unfair test and may simply assess the capacity to write a decent essay in praise of self and not capture anything real about potential as a nurse.**
**Content validity**	
**Construct validity**	
***Unitary Concept of Validity***	
**Credibility**	**The PS given its uncertain derivation is not a reliable source of information and this is compounded by loosely defined criteria and subjective interpretation by the marker.**
**Fitness**	**Intuitively the criteria appear relevant, but they are broad and general concepts without clear definition, developmental history, or other justification.**
**Robustness**	**Procedure and calculations are systematic, but it is difficult to be sure of a valid estimate of a candidate’s ability since no attempt can be made when marking the PS to deal with breaches of the assumption of genuine self-report made evident here.**
**Reliability**	**Item reliability: the coefficient of reliability (internal consistency) is acceptable, alpha = 0.77 (HEI 1) 0.78 (HEI 2). Invigilation is not a requirement and there is otherwise no attempt to ensure external consistency of PS completion under similar conditions so undermining dependability. There are several variables confounding use: the UCAS PS may be written for several Providers, sources of guidance and support are many and varied and access to it uneven, the concepts being tested are not well defined and subjective interpretation of the concepts and evaluation of the personal statement make for less reliable standards of marking.**
**Integrity**	**The PS is a free text open response so potentially genuine honest and moral: but advice and assistance are readily available with expert advice, if it can be afforded, so uncertainty exists about the true voice of the applicant while marking appears to assume an applicant to be sole author.**
**Representativeness**	**Standardisation of process and representativeness of the PS is undermined by modest validity and reliability; scores are ordinal not interval or ratio scale, so distributions of scores are layered creating multiple ties when rank ordering candidates.**
**Coherence**	**The broad and general concepts used as criteria for marking the PS are interpreted subjectively and scored differently by tutors: coherence is undermined.**
**Transparency**	**Information about the candidate comes to an unknown extent from co-authorship and guidance of variable quality from other people on how to make a good impression.**

PS procedure and calculations are systematic but scoring and differentiation of candidates lacks credibility and consistency due to the ordinal nature of the scoring, subjectivity in marking, and doubts around authorship.

### PS: reliability and errors of measurement

The internal reliability of the PS was estimated using Cronbach’s Alpha and item reliability was found to be acceptable. (See Table 3 in which HEI results are compared with the NM results).

**Table 3:** Coefficient of reliability of PS and NM test items

	NM 2018 (***n*** = 228)	NM 2015 (***n*** = 63)	HEI 1 PS^**a**^ 2018 (***n*** = 196)	HEI 2 PS^**a**^ 2018 (***n*** = 137)
**Cronbach’s alpha**	**0.9449**	**0.9437**	**0.7769**	**0.7655**

^a^Some applicants applied to both HEI 1 and HEI 2 see Table 4 below

A sub-group of applicants (*n* = 132) were ‘double marked’ blind and despite the internal reliability of the test, considerable variance in marking between HEI tutor teams became evident (Table 4).

**Table 4:** PS texts double marked blind at two HEIs: descriptive statistics

	PS-HEI 1	PS-HEI 2	Comment
**N**	**132**	**132**	**132 applicants applied to both HEIs**
**Mean**	**76.09**	**68.99**	**PS HEI 1 > mean: substantial difference and effect size [Cohen’s d = 0.44]**
**SD**	**14.38**	**17.4**	**PS HEI 1 Tighter spread**
**Median**	**75.00**	**68.75**	**PS HEI 1 Higher mid score**
**Mode (no. at mode)**	**75.00 (30)**	**75.00 (18)**	**PS HEI 1 Flatter mode (more scores tied at mode)**
**Minimum**	**18.75**	**25.00**	
**Maximum**	**100**	**100**	
**Skewness**	**−0.76**	**−0.44**	**PS HEI 1 easier to score on; > − ve. skew (see** Fig. 2**)**
**Kurtosis**	**1.45**	**−0.35**	**PS HEI 1 peaked: HEI 2 flatter (see** Fig. 2**)**

**Fig. 2:**
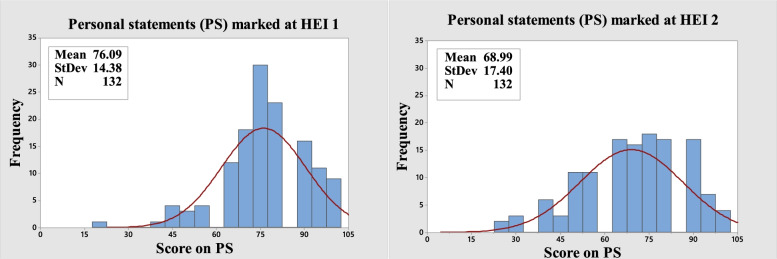
Histograms (with normal distributions): the same PS scripts marked at HEI 1 and HEI 2

There was a low correlation coefficient (*r* = 0.27) statistically significant at *p* = 0.002 (alpha = 0.05). ‘Agreement’ was low (only 5 < 10% variance between scores was explained). The histograms, and individual value plots in Figs. 1 and 2, show there is a greater negative skew in HEI 1 scoring suggesting easier marking. The higher mean and median of HEI 1 data also indicates easier marking.

**Fig. 3:**
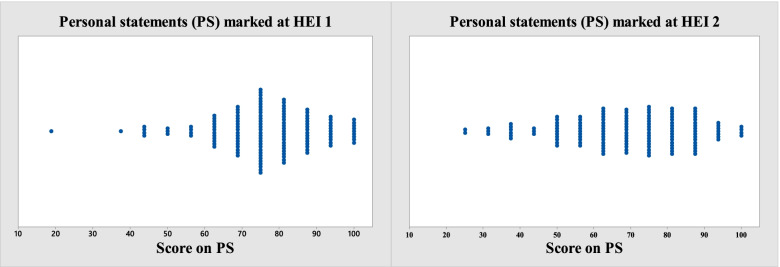
Individual value plots: the same PS scripts (*n* = 132) marked at HEI 1 and HEI 2

The PS criteria marks are ordinal numbers (‘categories’ 0 to 4). The overall score on the PS is the sum of the numbers on the four criteria. Ordinal numbers do not represent quantities or counts they represent rank positions in a group. They tell us nothing about distances between ranking positions. The effect of this on selection can be seen in Figs. 2 and 3 below where many ties can emerge.

These findings indicate that while the internal reliability of PS and its criteria (item) scores is acceptable considerable ‘error’ in the sense of bias or inconsistency can arise in the scoring of the scripts between tutor markers and teams of tutor markers.

### PS: Fairness

According to APA test standards, the testing or assessment process should be standardised to ensure that test-takers receive fair and equitable treatment during all phases of the testing or assessment process [2]. This is true of the PS only in the sense that all applicants to an HEI are set the same task and the outcome is marked according to a common scheme.

The research found that fairness was undermined by uneven marking standards and that levels and standards of advice and assistance in writing up the PS varied greatly between schools of origin and between pupils.

### Evaluation and comparison of PS and NM as psychological tests

#### Test standards and psychometric properties

The psychometric limitations of the PS are summarised in Table 5 as weak discriminatory power, questionable reliability in marking, inconsistency in administration, and concern about what is measured. A comparison is made with the acceptable standards of NM.

**Table 5:** Comparison of basic psychometric characteristics of the NM and PS scales

Criteria	Nurse Match	Personal Statement
**Discriminatory power**	**Continuous measure (*no ties*)**	**Ordinal ranking (*into a few categories)***
**Reliability /Consistency of use and marking**	**High item reliability / High consistency**	**Acceptable / Questionable**
**Validity /Does it measure suitability for nursing?**	**Yes / Measures suitability in a consistent manner using professional nursing values**	**Yes /Text marking is a subjective process and suitability is assessed using broad and general characteristics**
**Standardised on a population / invigilated, with consistent admin and immediate scoring?**	**Yes / Yes, invigilated, with consistent admin and objective automated scoring.**	**Yes / Completion unsupervised, support unconstrained, scoring is subjective, not automated, and so inconsistent**

When the quality of assessment was considered using basic psychometric principles, it was found that the PS had limitations concerning validity, consistency, standardisation, and scoring including a non-normal distribution of scores (Table 6). In summary, it was found that the PS does not meet international psychometric standards for psychological tests [2].

**Table 6:** Research findings for psychometric properties of PS and NM

Psychometric Principle	Personal Statement (PS)	Nurse Match (NM)
**Validity**	**Validity is questionable: face validity is fine, but concerns exist about content, construct, and other elements of validity.**	**Evidence supports acceptability of content, construct, and unitary concepts of validity**
**Reliability**	**Acceptable internal reliability (Cronbach’s alpha 0.77 HEI 1: 0.78 HEI 2)**	**Excellent internal reliability: (Cronbach’s alpha 0.94)**
**Consistency**	**Responses indirect and unsupervised, criteria are not well defined, and scoring is subjective, and inconsistent inter-marker**	**Responses are direct, value criteria clear and scoring is automated.**
**Standardisation**	**Administration is standardised and systematic but there are issues with test completion and marking**	**Administration is invigilated standardised and systematic and includes test completion and immediate scoring**
**Scaling, norming, and scoring**	**No direct response scales. Text scored subjectively. 5 category (0–4) ordinal scores on criteria. Scores cluster, many tied scores occur, distribution non- normal.**	**SDS scale. Direct response, automated score calculations. Interval score distributions typically approximate normality, tied scores highly unlikely.**

### Descriptive statistics and test characteristics

Test norms are set out in Table 7 and show that applicants found it easier to score on the PS than NM (PS mean scores all ~ 70.00 against NM mean 47.82 and all PS skews are negative against the NM positive skew). PS scores clustered around the mode (clusters of between 18 and 38 applicants) while NM scores did not, and clustering created PS distributions of scores that were non-normal while NM created a quite symmetrical normal distribution. Internal consistency was acceptable for PS but much more desirable for NM in terms of confidence about discriminations between applicants (PS; α = ~ 0.75: NM; α = 0.945).

**Table 7:** PS scores marked at HEI 1 and HEI 2 and NM comparison: descriptive statistics

Applicants	***(n = 196)***	***(n = 137)***	***Same PS (n = 132)***		***(n = 228)***
	PS HEI 1	PS HEI 2	PS HEI 1	PS HEI 2	NM
**Mean**	**74.62**	**68.8**	**76.09**	**68.99**	**47.82**
**SD**	**16.22**	**17.56**	**14.38**	**17.40**	**13.98**
**Median**	**75.00**	**68.75**	**75.00**	**68.75**	**46.86**
**SE Mean**	**1.16**	**1.50**	**1.25**	**1.51**	**0.926**
**Mode**	**75 (38)**	**75 (19)**	**75 (30)**	**75(18)**	*
**Skewness**	**−0.77**	**−0.43**	**−0.76**	**−0.44**	**0.24**
**Kurtosis**	**1.42**	**−0.42**	**1.45**	**−0.35**	**−0.16**
**AD statistic**	**3.650**	**1.45**	**1.894**	**1.402**	**0.55**
***P*value**	**< 0.005**	**< 0.005**	**< 0.005**	**< 0.005**	**0.159**
**Normal Distribution**	**No**	**No**	**No**	**No**	**Yes**
**Cronbach’s alpha**	**0.7769**	**0.7655**	**0.7514**	**0.7563**	**0.9449**

### The PS scoring process compared with NM

A key finding was that both validity and reliability of the PS were undermined by lack of rigour in the marking process. See Table 4 for differences in the statistics describing marking of the same scripts by two HEIs. Scoring in NM is automatic and internal reliability is high (α = 0.945).

Considerable ‘error’ in the sense of subjective or systemic sources of difference arose in the marking of the PS and is most clearly seen in and Figs. 1 and 2. It seems reasonable to infer that the problem may be a general one for the PS. This potential source of error is not present in the NM scoring process.

### ‘Suitability for nursing’: meaning of the concept

Factor analysis was used to help explicate how PS and NM assess the concept ‘suitability for a career in nursing’. Both appear to be unidimensional tests – there is one stable eigenvector, ‘Factor 1’, that explains most variance on each PS and NM data item. This factor has been called ‘suitability for nursing’ – but the meaning of the concept is distinctly different for the PS and NM as indicated by the data items columns in Table 8.

**Table 8:** Factor loadings on item variables (eigenvalues)

Data Items NM	NM Factor 1	Data items PS ***(HEI 1 and HEI 2)***	PS HEI 1 Factor 1	PS HEI 2 Factor 1
**Person Centredness**	**0.844**	**Desire for career**	**0.82**	**0.81**
**Accountability**	**0.984**	**Motivation**	**0.823**	**0.853**
**Trustworthiness**	**0.924**	**Professional Expectations**	**0.667**	**0.742**
**Integrity**	**0.860**	**Decision making**	**0.726**	**0.708**
**Commitment to personal development**	**0.749**			
**Teamworking**	**0.865**			
**VARIANCE**	**4.5833**		**2.32**	**2.43**
**% common variance**	**0.764**		**0.58**	**0.61**

In short PS assesses an applicant’s ‘Suitability’ in terms of attitude to nursing as a career while NM measures their inherent nursing values against professional values.

### Demand on resources

Having considered both tests, in the light of the experience of using the PS, the finding was that the PS would make greater demands on staff resources and be less cost-effective than NM: Table 9.

**Table 9:** PS and NM: demand on School of Nursing resources

	PS	NM
**Demand on staff resources**	**Quite high. Tutors marking hundreds of texts with due care plus internal admin is resource intensive.**	**Low demand. Admin and marking (with quality control) is external. There are options to defer costs.**

## Discussion

The aim of this study was to evaluate the PS as a shortlisting tool for admission to Nursing within two HEIs in the UK and in the process compare it with NM, a psychological test with established standards. Interestingly the study found that both the PS and NM are useful tests of suitability for shortlisting to interview for nursing programmes. However, they attribute potential suitability to different characteristics and there are concerns about the psychometric probity of PS that were not present with NM. Although there have been many studies that have examined the reliability and validity of admissions tools used to select students there is a paucity of research on the use of the Personal Statement. This study provides evidence to show that the PS has poor reliability both for the statement itself and also poor inter-rater reliability amongst the assessors. Similar studies examining the reliability of the PS have been previously reported most notably by Patterson and Roberts et al. [16] however studies citing the issues around internal marker reliability are less prevalent in the literature. The psychological tool used in this study is a bespoke values-based tool which measures personal nursing values against professional values whose provenance is clear. The PS assesses mindset in sensible, practical but general terms whose definition and derivation are not clear. Psychological tools such as NM are used within some institutions however their application to nursing is less evident with limited research in this area.

The PS assesses mindset in sensible, practical but general terms whose definition and derivation are not clear. The writing of a PS creates knowledge of unknown reliability, with anticipated plagiarism or coaching and subjective scoring. Subjectivity in marking the PS was evident from the disconcerting difference found between sets of tutor markers scoring the same tests. The subjective or systemic bias is made possible by the form of the PS ‘test’ as an essay; NM data responses are direct and spontaneous, and scored automatically.

Given the difference in what is being assessed and how effectively this is being done, it is not surprising that there is almost no correlation statistically speaking between scores by the same individuals on PS and NM (*r* = 0.111 *P*-Value = 0.199: alpha = 0.05). This implies a decision about what potential characteristics are to be tested for and how this might be done most effectively.

In summary, the findings provide practical, theoretical, statistical, and qualitative reasons for concluding that the PS has unacceptable limitations as a measure of suitability, does not compare well with international test standards, and would be hard to defend. It is hoped that the results reported in this paper will contribute to the literature on the use of Personal Statements in selection and additionally will encourage further empirical studies on the use of psychological tests that provide a cut score which institutions can confidently use to make decisions for access to nursing programmes. It is incumbent on those charged with selection to provide a fair and equitable admissions process, not only to maintain quality assurance standards, but also to ensure that the best people with the right values are admitted to nursing programmes.

There is also the issue of appeals to be considered and the merit of having reliable tests to support the decision-making process is something that is likely to be welcomed by university governing bodies. An additional and equally important point is the economic and indeed moral implications of employing a system that has been proven to be unreliable and upon which life changing decisions for the applicant are made, particularly in the current climate where the efficient use of both academic and administrative resources is paramount.

### Limitations

The fact that the sample was self-selecting and non-probability-based imposes limitations on what we can infer about the annual cohort of applicants, but the number of participants (*n* = 228) is sufficient to permit sound inferences to be made about the practical application and psychometric properties of the tests.

## Conclusion

Identifying the best selection tool is a priority for all oversubscribed nursing programmes, who want to train high quality graduates to enter the workforce. These findings however illustrate that the PS does not meet international psychometric standards for psychological tests [2]. Other concerns on the effectiveness of personal statements for selection have been raised in the literature [5, 14, 15].

The results of this study can be used by nursing education policy makers to inform on the continued use of the Personal Statement for admission to nursing. The evidence in this study not only highlights the serious shortfalls of the Personal Statement but also demonstrates the credibility of an effective psychological test (NM) as a viable alternative to the Personal Statement. The psychological test reported in this study has been shown to meet the essential criteria of any selection methodology i.e. it is standardised, reliable, valid and fair. It can therefore be concluded that Nurse Match offers a robust and valid alternative to the nursing personal statement as a method for determining the suitability of applicants for a career in nursing and shortlisting for interviews.

## Data Availability

The data sets to support this manuscript is available at the following link: https://www.qub.ac.uk/schools/SchoolofNursingandMidwifery/FileStore/Filetoupload,945100,en.pdf.
